# Pemphigoid diseases in patients with end-stage kidney diseases: pathogenesis and treatment

**DOI:** 10.3389/fimmu.2024.1427943

**Published:** 2024-07-10

**Authors:** Liu-Yi-Yi Yang, Yu-Lu Wang, Ya-Gang Zuo

**Affiliations:** ^1^ Department of Dermatology, State Key Laboratory of Complex Severe and Rare Diseases, National Clinical Research Center for Dermatologic and Immunologic Diseases, Peking Union Medical College Hospital, Chinese Academy of Medical Sciences and Peking Union Medical College, Beijing, China; ^2^ Department of Dermatology, Xiajin Country People’s Hospital, Dezhou, Shandong, China

**Keywords:** pemphigoid diseases, bullous pemphigoid, end-stage kidney disease, mechanism, treatment

## Abstract

**Background:**

Pemphigoid diseases constitute a group of autoimmune blistering disorders characterized by subepithelial blistering. The association between pemphigoid diseases and both end-stage kidney disease (ESKD) and its treatment is notable. However, there is limited evidence about the management of pemphigoid diseases in patients with ESKD. This systematic review compiled case reports and relevant studies, summarized the underlying mechanisms of pemphigoid diseases in patients with ESKD, and summarized the efficacy of various therapies.

**Methods:**

A systematic search of PubMed and Embase was performed for articles published between 1982 to June 2, 2024.

**Results:**

Fifty-three case reports and eight relevant studies were included. Triggers for pemphigoids in patients with ESKD included materials used to treat ESKD, immune dysregulation of patients with ESKD, and rejection of renal allograft. Treatment for these patients included removing triggers, as well as administering of corticosteroids, mycophenolate mofetil (MMF), tetracyclines, rituximab, methotrexate, dapsone, azathioprine, cyclosporine, intravenous immunoglobin (IVIG), plasmapheresis, and Janus kinase inhibitors.

**Conclusion:**

Removing triggers is the most effective strategy. Despite their suboptimal efficacy, corticosteroids remain the most commonly used agents in this patient population. MMF, tetracyclines, and rituximab are less used but with benefits. There are significant adverse effects associated with methotrexate treatment. Other treatment may also be beneficial and require further investigation. These findings may enable clinicians to optimize the therapeutic approach for these patients.

## 1 Introduction

Pemphigoid diseases are a spectrum of autoimmune blistering dermatoses comprising bullous pemphigoid (BP), mucous membrane pemphigoid (MMP), p200 pemphigoid, epidermolysis bullosa acquisita (EBA), and linear IgA dermatosis (LAD). Among them, BP is the most common subtype, with an incidence rate of 34.2 (95% confidence interval 19.2-60.7 per million person-years) (1). BP patients develop autoantibodies against BP180 and/or BP230, which are crucial proteins within the dermal-epidermal junction (DEJ) (2). MMP predominantly affects mucous membranes, especially the mouth and conjunctivae (3). Autoantibodies typically target different autoantigens such as BP180, laminin 332, and BP230 (3). Similar to BP clinically, p200 pemphigoid is characterized by autoantibodies against the 200-kDa protein of the DEJ (4). EBA is characterized by autoantibodies against collagen VII, and can present in classic or inflammatory subtype (4). The classic subtype typically exhibits skin fragility and bullous lesions at trauma-prone areas, while the inflammatory subtype resembles BP or MMP (4). LAD, the most prevalent pemphigoid disease in children, is distinguished by linear deposits of IgA at the DEJ (4).

Comorbid conditions, such as end-stage kidney disease (ESKD), have been reported in patients with pemphigoid diseases. ESKD occurs when dialysis or renal transplantation is essential to maintain patients’ survival (5). The relationship between BP and ESKD, as well as its treatment, has been demonstrated in dozens of studies. Patients on dialysis are more susceptible to cutaneous conditions, including pemphigoid diseases. BP has been increasingly observed in these patients. Morimoto et al. presented several BP cases in patients receiving peritoneal dialysis (PD), suggesting a potential correlation between the dialysis process and the onset of BP (6). This relationship was further supported by J Miao et al., who reported a patient on hemodialysis had levofloxacin-induced BP, indicating the potential role of certain medications in triggering BP among patients with ESKD (7). A nationwide population-based cohort study revealed that the hazard ratio (HR) for BP in patients with ESKD was 2.12 compared to individuals without chronic kidney disease (CKD) (8). Similarly, another study identified CKD as a significant risk factor for BP, with dialysis-dependent patients showing the highest risk (9). A case-control study of 91 BP cases found that BP significantly increased the odds of comorbid ESKD (adjusted odds ratio: 3.82) (10). Studies also demonstrated that the incidence of BP among patients on dialysis was much higher than that observed in the general population (6, 11).

Managing pemphigoid diseases in patients with ESKD can be challenging due to their compromised renal function, the administration of immunosuppressants (IS), and altered immune response. These patients require treatments that can effectively manage symptoms without exacerbating the underlying renal condition. However, there is a scarcity of research focusing on the management of pemphigoid diseases among patients with ESKD. This systematic review aims to summarize all reported cases with a definitive diagnosis and provide guidance to clinicians regarding appropriate treatment methods under different circumstances.

## 2 Methods

### 2.1 Search strategy

**Figure 1:**
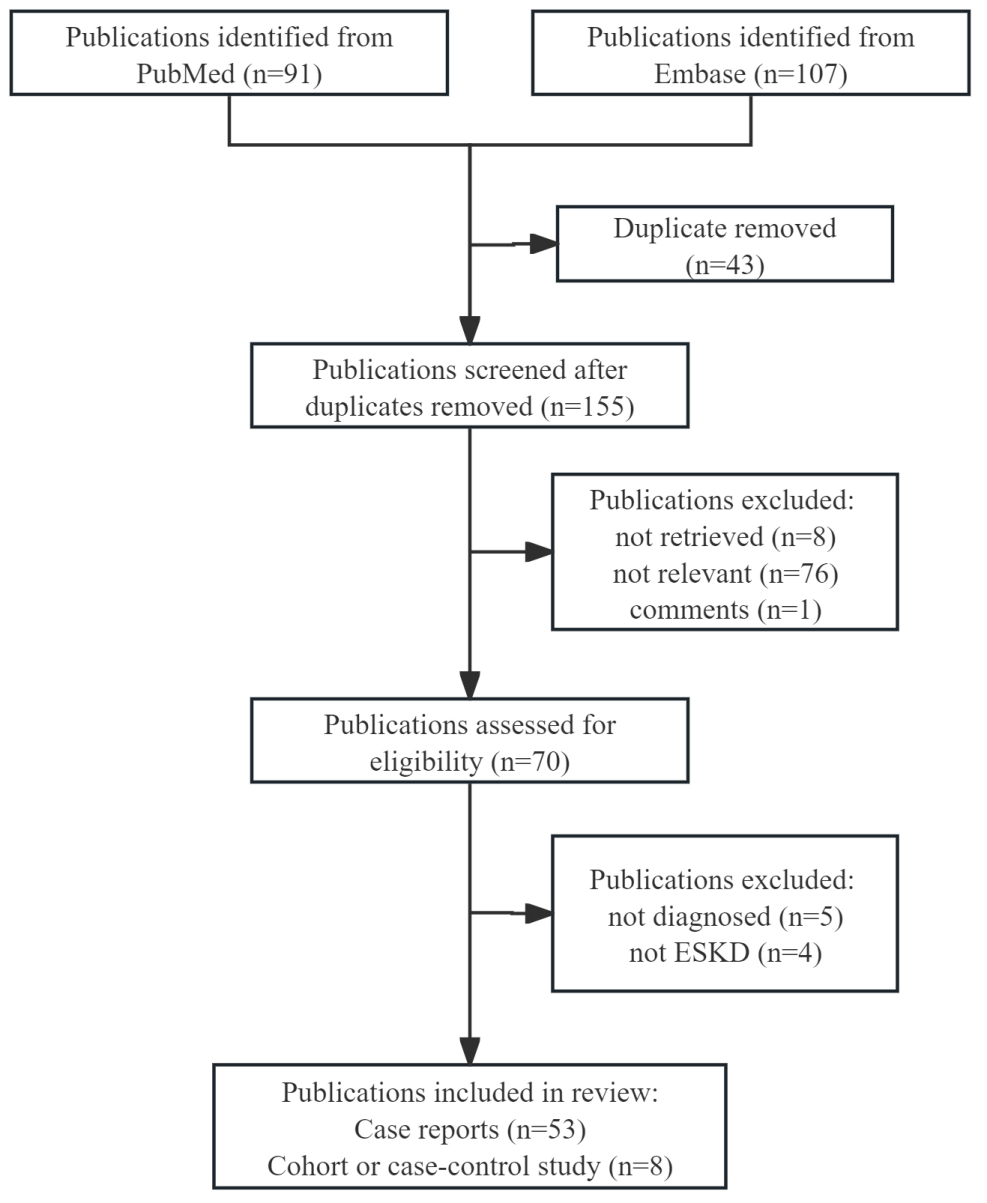
PRISMA flow diagram. ESKD, end-stage kidney disease.

A literature search was performed using PubMed and Embase from 1982 to June 2, 2024. The search terms included pemphigoid, epidermolysis bullosa acquisita, linear IgA bullous dermatosis, end-stage kidney disease, kidney transplantation, and dialysis. This study was conducted under the guidelines of the Preferred Reporting Items for Systematic Reviews and Meta-Analyses (PRISMA) and a PRISMA flow diagram is shown in **Figure 1**.

### 2.2 Eligibility criteria

Case reports were included if pemphigoid diseases were diagnosed based on at least two of the following conditions (1): subepidermal blister (2), positive direct immunofluorescence results (linear IgG or complement component C3c staining for BP) (3), positive indirect immunofluorescence results (linear deposits of IgG at the DEJ, or IgG staining on the epidermal side of salt-split skin for BP), or (4) positive serum autoantibodies (anti-BP180 antibodies (+) and/or anti-BP230 antibodies (+) for BP). Considering the impaired clearance of drugs in targeted patients, drug-induced pemphigoid diseases in patients with ESKD were included. Exclusion criteria included non-original publications and case reports not meeting the inclusion criteria.

### 2.3 Statistical analysis

Data were presented as an absolute number with percentage for categorical variables and mean with range for continuous ones. Descriptive statistics were used to summarize the characteristics of patient populations. The effectiveness of different treatment options was evaluated based on the reported clinical response rates.

## 3 Results and discussion

### 3.1 Study identification and patient characteristics

Fifty-one cases of the onset of BP in patients with ESKD and nine cases of other pemphigoid subtypes have been published (**Supplementary Tables 1, 2**). Apart from one patient whose sex was not described, the female-to-male ratio in patients with ESKD and concurrent BP was 0.52:1, lower than that of patients with BP (1.87:1) and patients with ESKD (0.71:1) (12, 13). Therefore, the reason behind male predominance in patients with concurrent ESKD and BP cannot be fully explained by male predominance in ESKD. Sex and sex hormones may have some roles on this comorbidity. The mean age was 50.5 years, also lower than the mean age of general patients with BP (74.2 years) and patients on dialysis (65 years) (14, 15).

### 3.2 Pathogenesis of pemphigoid diseases in patients with ESKD

Pathogenesis of pemphigoid diseases in patients undergoing dialysis is a multifaceted process influenced by various factors, though the precise mechanisms are not yet fully understood. Materials-induced hypersensitivity, immune dysregulation, and medication-induced immunosuppression are believed to be key contributors to their development.

#### 3.2.1 Materials-induced pemphigoid diseases

**Figure 2:**
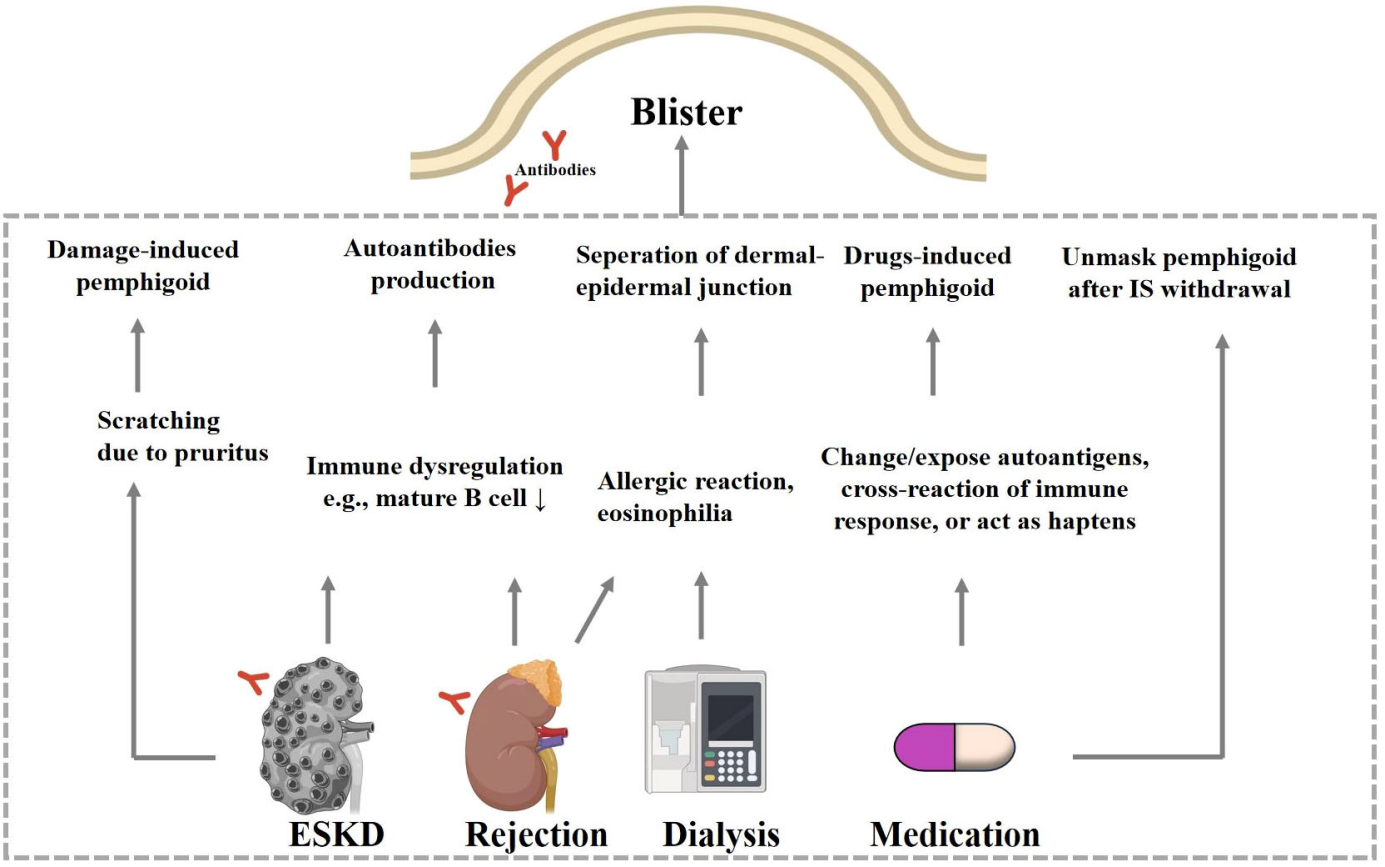
Hypothesized mechanisms of ESKD-associated pemphigoid diseases. Patients with ESKD develop pemphigoid diseases via skin damage due to scratching and via autoantibodies production due to shared autoantigens in kidney and skin. Renal allograft rejection is also accompanied by immune dysregulation. Materials used during the dialysis process may cause eosinophilia via hypersensitive reaction, and these eosinophils may then infiltrate to the BMZ and result in dermo-epidermal separation. When the dose of immunosuppressant or other drugs is adjusted. ESKD, endstage kidney diseases; IS, immunosuppressant.

Materials commonly associated with the induction of BP during dialysis include prosthetic vascular grafts, PD catheters and dialysis membranes. Vascular grafts and PD catheters serve as access points for dialysis. The initial lesions of fistula-triggered BP are typically located around the fistula site, as evidenced by eleven cases (6, 16–23). In one patient with BP, bullous lesions were limited to his hand distal to the fistula (24). Two patients experienced BP after changing the dialysis membrane from a polymethylmethacrylate membrane to a cellulose triacetate membrane or rinsing of dialysis circuit (25). These materials may induce BP via an allergic reaction and subsequent eosinophilia in the bloodstream (21, 25). BP is associated with blood eosinophilia, since 50%-60% of patients with BP exhibit blood eosinophilia (26). At the same time, blood eosinophilia is not uncommon in patients with ESKD, accounting for 5% of patients with dialysis and 20%-36% transplant patients with acute allograft rejection (27). Mutsuyoshi et al. reported three hemodialysis patients with idiopathic hypereosinophilia syndrome, which is characterized by blood eosinophilia and damage to multiple organs including skin (28). Therefore, eosinophils of patients with blood eosinophilia may infiltrate into skin. Eosinophils may contribute to pemphigoid diseases by forming eosinophil extracellular traps and releasing toxic proteins, which cause the separation of DEJ (29, 30) (**Figure 2**). These observations indicate a potential link between dialysis-related factors and the onset of pemphigoid diseases, possibly through exposure to foreign antigens and immune dysregulation. Moreover, 40%-84% of patients on hemodialysis exhibit pruritus (31, 32). The pathogenesis of uremic pruritus may be related to dry skin, higher dermal number of mast cells and lower clearance of pruritogenic molecules (33). Skin damage caused by frequent plaster removal for medication and scratching due to pruritus further contributes to the development of BP (18). Therefore, it is crucial to assess and manage pruritus appropriately in these patients. Patient-reported outcome tools and other effective measures may help clinicians to monitor pruritus and avoid scratching-associated skin conditions (34).

#### 3.2.2 Medication-induced pemphigoid diseases

Studies have also suggested a potential association between medication use in patients with ESKD and pemphigoid diseases. IS may mask pemphigoid diseases because of its anti-autoimmune nature. After tapering IS, a significant number of autoantibodies may be generated and contribute to pemphigoid diseases. This is evidenced by nine patients who developed pemphigoid diseases after decreasing or withdrawing of IS (35–43). Resolution of BP after restarting IS therapy has been observed. The average interval between medication changes and pemphigoid diseases was 4.48 months (range: 4 days-12 months) in these patients (excluding one case with unclear data). Furthermore, drugs such as tacrolimus have been shown to reduce the population of regulatory T cells, subsequently leading to increased autoantibodies production (44). Substituting tacrolimus with corticosteroids and mycophenolate mofetil (MMF), which inhibits both cellular and humoral immunity, helped alleviate BP (45). Two BP cases were induced by mammalian target of rapamycin (mTOR) inhibitors, as evidenced by immediate resolution upon drug discontinuation followed by relapse after rechallenge (46). Other agents, such as cefixime and levofloxacin, were also considered to induce pemphigoid diseases in patients with ESKD (7, 47). Mechanisms of drug-induced pemphigoid diseases included the possibility that certain drugs could change the structure of autoantigens, expose previously sequestered autoantigens, promote autoantibodies production by acting as antigenic haptens, disrupt the DEJ directly, and cause cross-reaction of the immune response (7, 47).

#### 3.2.3 Rejection-associated pemphigoid diseases

Thirteen cases associating BP with kidney transplant rejection have been reported (23, 36, 37, 40, 41, 43, 48–54). Among them, most were chronic rejection, with only four cases having acute rejection (36, 37, 51, 54). Mucosal involvement has been observed in two patients (48, 52). One patient developed pemphigoid disease with autoantibodies to both laminin γ1 and γ2 subunits during renal transplant rejection (42). Immune dysregulation during rejection response may contribute to pemphigoid diseases. Interestingly, in post-transplantation patients who have normal graft function, a broad array of autoantibodies can also be generated, which may explain why pemphigoid occurs in these patients (55). Furthermore, genetic predisposition and environmental triggers such as infections or trauma may play a role in pemphigoid pathogenesis in patients with ESKD.

#### 3.2.4 Immune dysregulation of patients with ESKD

Pemphigoid diseases and ESKD may have a shared pathogenesis. First, components shared by the basement membrane zone (BMZ) and the glomerular basement membrane (GBM), acting as autoantigens, may induce autoantibodies. For instance, two cases of pemphigoid diseases comorbid with Alport syndrome, a genetic kidney disease characterized by abnormal type IV collagen, were reported (56, 57). Type IV collagen is typically localized to the base of BP blisters. The specific autoantibodies towards the α-5 chain of type IV collagen have been described to cause both subepidermal blisters and renal insufficiency (58). These autoantibodies were also found in rats experiencing renal allograft rejection, explaining the association between rejection and BP (59). One case with BP comorbid with anti-GBM disease also provided evidence (60). Second, the dysregulation of the immune system in patients on dialysis may lead to aberrant autoimmune responses, contributing to the pathogenesis of pemphigoid diseases. Loss of NK and mature B cell subset, as well as an increase of a proinflammatory monocyte subset has been observed in severe CKD (61).

Overall, these findings underscore the complexity of pemphigoid pathogenesis in patients with ESKD and highlight the need for further research to elucidate the underlying mechanisms. Understanding these mechanisms may provide insights into potential therapeutic targets and strategies for managing pemphigoid diseases in this vulnerable population.

### 3.3 Treatment

#### 3.3.1 Triggers removal

As described previously, triggers of pemphigoid in patients with ESKD include materials and drugs used in treatment. Removing prosthetic vascular graft, prerinsing the dialysis circuit with dexamethasone solution (5 mg/L), changing dialysis membrane or disinfectant, and discontinuing inducing drugs may benefit patients (6, 21, 24, 25, 46, 62, 63). In our studied population, BP-associated drugs include furosemide, levofloxacin, cefixime, everolimus, and sirolimus. Additionally, other BP-inducing drugs, including gliptins and PD-1/PD-L1 inhibitors, should be considered (64). While most patients manifested symptoms relatively quickly after the presence of triggers, three patients had symptoms even after triggers had presented for years (18, 22, 63). Signs of allergic response or rejection response also suggest the presence of triggers.

The transplanted kidney is another trigger for pemphigoid diseases. In cases where patients are refractory to other treatments, renal graft removal may be necessary. Thirteen cases were resolved without recurrence after nephrectomy, with relevant antibodies disappearing in two cases (21, 23, 36–40, 48, 50, 52, 65). One case was resolved after graft atrophy (66). Although most cases benefited from nephrectomy, the condition of BP may worsen because of a sustained post-nephrectomy immune response (36). To avoid this situation, it may be necessary to continue IS therapy for a certain period after nephrectomy.

#### 3.3.2 Corticosteroids

In the realm of dermatology, the effectiveness of corticosteroids in treating pemphigoid diseases is well-established and corticosteroids remain a mainstay treatment. Studies have shown the effectiveness of corticosteroids in managing BP, even in complex cases like post-transplant recipients or in patients experiencing kidney transplant rejection (35, 41). Among 59 reported cases using corticosteroids, 14 (23.73%) patients were recalcitrant (23, 25, 37, 44, 46, 48, 50, 52, 62, 67–70). The dosage of corticosteroids was reported in twenty-two cases who responded well. Among them, oral prednisone was initiated at 5-75 mg/day with an average of 44.71 mg/day. In cases reported by dose per kilogram, oral prednisone was initiated at 0.5-1.5 mg/kg/day with an average of 0.94 mg/kg/day. This starting dose is higher than the recommended dosage of 0.5 mg/kg/day according to the European Academy of Dermatology and Venereology (71). Resolution typically can be observed within weeks, as reported in five cases. Among recalcitrant patients, two cases only used topical steroids, and others used prednisolone with the average daily dose of 50 mg. Triggers removal benefited patients who responded minimally to corticosteroid, suggesting the importance of triggers identification. After tapering or stopping the dose of corticosteroids, five patients relapsed (38, 39, 50, 72, 73). Severe infection was reported in one case (6).

Corticosteroids treatment of pemphigoid diseases in patients with ESKD requires additional caution. The importance of individualized dosing and vigilant monitoring for adverse effects should be highlighted, due to the renal impairment and altered drug metabolism of patients. To help reduce the dosage of systemic corticosteroids and minimize the risk, adjunctive therapies such as azathioprine, MMF, and biological agents, may be considered.

#### 3.3.3 Dapsone

Dapsone, an antibiotic with anti-inflammatory properties, has also been a mainstay in treating pemphigoid diseases and a second-line chemoprophylactic drug for *pneumocystis* pneumonia in solid organ transplant recipients. However, specific studies on the use of dapsone in patients with concurrent ESKD and pemphigoid diseases are limited. This kind of treatment was reported in eight cases and benefited four patients with an average reported dose of 62.5 mg/day (20, 72, 74, 75). Adverse effects including epigastric pain and mild hypertension were reported (73). It is worth noting that dapsone-induced methemoglobinemia (MHb) occasionally developed in renal transplant recipients. A cohort study found that 12/26 (46.15%) post-transplantation patients developed MHb after receiving dapsone (76). Therefore, dapsone treatment should be used with caution in patients with ESKD.

#### 3.3.4 Immunosuppressive agents

##### 3.3.4.1 Mycophenolate mofetil

MMF effectively suppresses both cellular and humoral immune responses, making it a potentially safe and efficient agent for managing pemphigoid diseases in patients with ESKD. Notably, MMF is a potent IS commonly used in organ transplant recipients (77). It has been used as an IS drug in nine patients with ESKD (16, 23, 35, 37–39, 41, 43, 51). However, the onset of BP has been observed in six cases after discontinuing MMF and other IS drugs. Three patients with ESKD used a combined therapy of MMF (at daily dose of 500 mg, 500 mg twice and 2,000 mg) and corticosteroids, and achieved resolution (42, 45, 70). A randomized clinical trial has demonstrated that MMF can halt the progression of immunoglobulin A nephropathy, a leading cause of ESKD in many countries (78). Therefore, MMF is a potential choice for the management of pemphigoid diseases in patients with ESKD, especially those who have undergone renal transplantation.

##### 3.3.4.2 Methotrexate

Methotrexate (MTX) is an inexpensive agent that can be used in pemphigoid diseases and renal allograft rejection. Only two patients with ESKD were treated with low-dose MTX for their BP, with a weekly dose of 10 mg and 5 mg, respectively (67, 68). Both cases developed life-threatening pancytopenia, indicating the toxicity of MTX in these patients. One patient died despite receiving calcium folinate and hemodialysis, while the other recovered after undergoing continuous veno-venous hemofiltration, receiving component blood transfusion, and receiving cytokine supportive treatment. Although a retrospective cohort study found that using low-dose MTX (5-10 mg/week) among patients with BP who have low renal clearance is safe, MTX should not be advised in patients with creatinine clearance less than 10 mL/min (79). Preexisting renal insufficiency and impaired renal MTX elimination can increase the risk of MTX toxicity (80). Therefore, MTX should not be prescribed to these patients.

##### 3.3.4.3 Cyclosporine

Cyclosporine was used as an anti-rejection drug in three patients with ESKD, and the onset of BP in two patients were observed during cyclosporine administration (49). Additionally, one patient developed BP after tapering cyclosporine (46). In one case, cyclosporine, in combination with corticosteroids, azathioprine and IVIG, failed to treat BP or suppress rejection (52). Although there is evidence to support the efficacy of cyclosporine in focal segmental glomerulosclerosis, which is a leading cause of ESKD (81), its benefits in patients with concurrent ESKD and pemphigoid diseases have yet to be assessed.

##### 3.3.4.4 Azathioprine

Azathioprine (AZA) has been utilized to prevent graft rejection in two patients with ESKD (49, 50). However, the onset of BP was observed in one patient after the discontinuation of AZA (50). Additionally, four patients with ESKD used AZA (50-100 mg/day for 2-4 weeks) in combination with other drugs to treat BP, with two achieving resolutions (41, 52, 82). The utilization of AZA was successful to reduce the reliance on corticosteroids in one patient (35). Therefore, AZA may be a safe and effective strategy to treat pemphigoid diseases in conjunction with renal allograft rejection, but further research is needed to validate its efficacy.

#### 3.3.5 Intravenous immunoglobulin

Intravenous immunoglobulin (IVIG), derived from healthy donors, is a blood preparation containing immunoglobulin and other components. It is recognized as a beneficial treatment in various autoimmune conditions and renal allograft rejection (83). The use of IVIG has also been shown to increase successful transplant rates in patients with ESKD (84). However, the use of IVIG presents therapeutic challenges and opportunities when treating pemphigoid diseases in patients with ESKD. IVIG has been reported to treat BP in three patients with ESKD, but all attempts were unsuccessful (52, 62). These patients ultimately recovered after the removal of triggers, underscoring the priority of identifying and removing triggers over drugs administration. One patient with concurrent LAD and ESKD achieved remission for more than one year by administrating IVIG (35 g/day for 3 days, repeated after 2-week intervals for the first 4 months, and repeated after 3-week intervals for the next 4 months) (73). Given its effectiveness on patients with ESKD, IVIG may offer benefits in managing pemphigoid diseases that occur during renal allograft rejection, as well as patients with pemphigoid diseases awaiting renal transplantation. However, the administration of IVIG requires careful monitoring for potential side effects such as fluid overload and acute renal failure. Further clinical trials are needed to evaluate its safety and effectiveness.

#### 3.3.6 Tetracyclines

Tetracyclines, either alone or in combination with nicotinamide, have been shown to benefit patients with pemphigoid disease (85). One patient with concurrent LAD and ESKD responded to tetracycline (2 g/day) and nicotinamide (1.5 g/day), but experienced severe diarrhea (73). Doxycycline, a second-generation tetracyclines, does not require dose adjustment when used in patients with renal impairments (86). Two of the three patients recovered by using doxycycline in conjunction with topical corticosteroids (18, 19), while the other was recalcitrant to doxycycline and other drugs, including systemic corticosteroids, dapsone, and niacinamide (44). A recent multicenter randomized controlled trial showed that doxycycline displayed a comparable efficacy in BP treatment to oral corticosteroids with minimized adverse events (87). These advantages make doxycycline a promising drug for managing of pemphigoids diseases in patients with ESKD. Although there was no report of minocycline treatment, similar benefits can be expected.

#### 3.3.7 Plasmapheresis

Plasmapheresis can be used to remove autoantibodies, immune complexes and cytokines that participate in pathogenesis of various autoimmune diseases, including pemphigoid diseases. Hence, it has emerged as a significant treatment option for these diseases. However, plasmapheresis failed to treat BP in two patients with ESKD and caused several side effects, including thrombocytopenia, coagulopathy, and sepsis. One of the patients recovered after the removal of a renal graft (44, 48). Both cases had prominent mucosal lesions concomitant exacerbating renal conditions, indicating the possibility of cross-reactive autoantibodies and underscoring the importance of removing responsible pathogenic factors. Albeit these abortive cases, the clinical efficacy of plasmapheresis cannot be denied. A large randomized controlled trial has exhibited the safety and effectiveness of plasmapheresis in patients with anti-neutrophil cytoplasm antibody-associated vasculitis, an autoimmune disease that can lead to renal failure (88). Additionally, a multicenter cohort study showed that plasmapheresis, together with IS, significantly improved renal survival rates in patients with anti-complement factor H-associated hemolytic uremic syndrome (89). Therefore, further exploration of how plasmapheresis performs in patients with pemphigoid diseases combined with ESKD is warranted.

#### 3.3.8 Biological agents

The importance of biological agents (such as rituximab, dupilumab, omalizumab, and mepolizumab) in the treatment of pemphigoid diseases has gained increasing recognition. A meta-analysis involving 296 patients with pemphigoid diseases showed that these drugs have benefits comparable to oral corticosteroids and are significantly safer (90). In our studied population, two patients receiving rituximab transfusions (for 2 or 4 infusions), and experienced symptom alleviation (37, 70). Rituximab, a B-cell depleting drug, has demonstrated efficacy in improving acute antibody-mediated renal transplant rejection (AMR), although its therapeutic effect in chronic AMR remains insignificant (91). Dupilumab has been utilized in nine patients with renal insufficiency as a safe and effective drug for various skin conditions, including atopic dermatitis, reactive perforating collagenosis, and uremic pruritus (92–95). Its potential use in patients with concurrent pemphigoid diseases and ESKD is promising. Additionally, it is worth mentioning that off-label use of tralokinumab, an anti-interleukin-13 antibody for treating atopic dermatitis, has successfully treated BP in a patient with ESKD (69). Further investigation is needed to assess the safety and benefits of these biological agents in the treatment of pemphigoid diseases in patients with ESKD.

#### 3.3.9 Janus kinase inhibitors

JAK inhibitors work by inhibiting the activity of one or more enzymes from the Janus kinase family, thereby disrupting the JAK-STAT signaling pathway, which plays a crucial role in immune response and inflammatory processes. A study by Brosius et al. illustrated the potential of JAK inhibitors as an alternative therapy for diabetic kidney disease (DKD), a condition often coexists with pemphigoid diseases and is the most common cause of ESKD (96). This result suggests that JAK inhibitors may also be effective in managing pemphigoid diseases in patients with ESKD. Moreover, the pharmacokinetics of tofacitinib, a JAK inhibitor, has been studied in patients with varying degrees of renal impairment (97). This is crucial for ESKD patients, as it enables individualized treatment strategies in managing pemphigoid diseases.

While the benefits are promising, the use of JAK inhibitors in patients with ESKD poses specific challenges. A phase two clinical trial on the efficacy of baricitinib, a JAK1/JAK2 inhibitor, in DKD, highlighted the necessity for tailored dosing and vigilant monitoring in such patients (98). Additionally, the risk of adverse effects, such as infections or anemia, could be heightened in these patients, necessitating a cautious approach and thorough risk-benefit analysis before initiating JAK inhibitor therapy. The results of a study by Sugawara et al. on the prediction of non-responders to JAK inhibitors in patients with rheumatoid arthritis further emphasized the importance of personalized medicine (99). This approach is also relevant for patients with ESKD, whose individual factors such as residual renal function, comorbidities, and concurrent medications must be considered.

Therefore, JAK inhibitors may be effective drugs for managing pemphigoid diseases combined with ESKD, yet it is imperative to approach their use with caution. The results from ongoing research and clinical trials will be key in further elucidating the efficacy and safety of these drugs in this patient population.

## 4 Conclusion

This review provides a comprehensive overview of pemphigoid in patients with ESKD, emphasizing the mechanisms and therapeutic strategies (**Table 1**). Gaining a better understanding of the pathophysiology of the pemphigoid diseases in patients with ESKD is essential for improving treatment strategies, allowing clinician to minimize invasive options. Overall, the identification and removal of triggers are the most important and effective approach for treating pemphigoid diseases in patients with ESKD. The relatively ideal drugs are MMF, corticosteroids, and biological agents (rituximab, especially). MTX is not recommended due to its severe adverse effects. Other treatment strategies, such as dapsone, azathioprine, cyclosporine, intravenous immunoglobulin, tetracyclines, plasmapheresis, and Janus kinase inhibitors, may be considered depending on individual circumstances. The use of these treatments in patients with ESKD must be approached with caution, considering the potential for altered drug handling and increased risk of adverse effects. Personalized treatment plans, careful monitoring, and a multidisciplinary approach are essential for the safe and effective management of pemphigoid diseases in this patient population.

**Table 1:** Evidence-based suggestion of pemphigoid therapy in patients with end-stage kidney disease.

Therapy	Number of cases	Response rate (%)	Effective Dosage	Suggestion
Nephrectomy	13	100	–	Recommendation
Other triggers removal	13	100	–	
Mycophenolate mofetil	3	100	500-2000 mg/day for 2 months	
Corticosteroids	59	76	>40 mg/day for 2 weeks, then taper gradually	
Rituximab	2	100	375 mg/m^2^/week for 2-4 weeks	
Tetracyclines (doxycycline)	3	67	100 mg twice a day, taper after 6 weeks and discontinue after 8 weeks	
Dapsone	8	50	50 mg/day	Cautious use
Azathioprine	5	60	50-100 mg/day for 2-4 weeks	
Cyclosporine	1	0	–	
Intravenous immunoglobulin	4	25	35 g/day for 3 days, repeated after 2-week intervals for the first 4 months	
Plasmapheresis	2	0	–	
Janus kinase inhibitors	0	–	–	
Methotrexate	2	0	–	Not recommended

## Author contributions

L-Y-YY: Visualization, Writing – original draft. Y-LW: Writing – original draft. Y-GZ: Conceptualization, Funding acquisition, Supervision, Writing – review & editing.
