# Supplementary material for: Pemphigoid diseases in patients with end-stage kidney diseases: pathogenesis and treatment
Source: Front Immunol. 2024 Jul 10;15:1427943. doi: 10.3389/fimmu.2024.1427943 (PMC11266006; doi:10.3389/fimmu.2024.1427943)
Supplement: Supplementary file 1 [file Table_1.docx]

**Pemphigoid diseases in patients with end-stage kidney diseases: Pathogenesis and treatment**

Liuyiyi Yang^1^, Yulu Wang^1^, and Ya-Gang Zuo^1^

^1^Department of Dermatology, State Key Laboratory of Complex Severe and Rare Diseases, National Clinical Research Center for Dermatologic and Immunologic Diseases, Peking Union Medical College Hospital, Chinese Academy of Medical Sciences and Peking Union Medical College, Beijing, China

**Correspondence**

Corresponding Author: Ya-Gang Zuo

No 1, Shuaifuyuan, Dongcheng District, Beijing, 100730, China

E-mail: zuoyagang@263.net, lilipumch2007@sina.com

ORCID: 0000-0002-2526-4331

**Supplement Table I. Reported cases of BP among Patients receiving dialysis**

| **No.** | **Age/sex** | **Latency** | **Characteristics** | **Therapy** | **Outcome** | **Ref** |
| --- | --- | --- | --- | --- | --- | --- |
| 1 | 56/M | 4 years after PD and furosemide use | Erythematous plaques on forehead, cheeks, and ear lobes; direct IF: linear deposits of IgG and C3 in basement membrane; immunoblotting: patient’s serum reacted with BP180 and BP230; anti-180 IgG (+), anti-BP230 IgG (+). | Oral prednisolone (10 mg/day), discontinue furosemide | Resolution | [1] |
| 2 | 62/M | 1 years after the initiation of PD | Pruritic blisters around the exit site of the PD catheter; anti-BP180 IgG (+); subepidermal blister; eosinophilic and lymphocytic infiltration, BP-specific autoantibody deposition. | Hydrocortisone-bacterial culture suspension mixture ointment for a month | Resolution | [2] |
| 3 | 72/M | 4 weeks after the induction of PD | Pruritic eruptions on hands, diffuse blisters on trunk and extremities; anti-BP180 IgG (+); subepidermal blister with eosinophils infiltration and BP-specific autoantibody deposition. | Oral prednisolone (60 mg QD for 2 weeks, then 10 mg BIW) | Lesions were improved but severe infection was caused. | [2] |
| 4 | 76/ND | 12 months after the Tenckhoff catheter was placed | Bullous, pruritic lesions around the exit site of the PD catheter; Anti-BP180 (+), anti-BP230 detectable; subepidermal blister with eosinophils infiltration; direct IF: linear deposits of IgG and C3 along the BMZ; indirect IF: IgG binding to the epidermal side of blister. | Systemic doxycycline (100 mg BID for 8 weeks), topical 0.05% clobetasol propionate (BID for 12 weeks) | Resolution within 2 weeks | [3] |
| 5 | 79/F | ND | Bullous, pruritic lesions with sero-hemorrhagic content, milia around the exit site of the PD catheter; Anti-BP180 IgG (+); epidermal spongiosis, subepidermal clefts with eosinophils infiltration; direct IF: linear deposits of IgG and C3 along the roof of the split. | Prednisone (0.5 mg/kg/die) and topical 0.05% clobetasol propionate (QD) for two weeks; dapsone (25 mg QD) and topical 0.05% clobetasol propionate (BIW) for maintenance | Resolution after 2-week therapy | [4] |
| 6 | 79/F | 5 years after the initiation of PD | Systemic diffuse pruritic blisters and eruptions; Anti-BP180 IgG (+); subepidermal blister with eosinophils infiltration and BP-specific autoantibody deposition. | Prednisolone (40 mg QD for 2 weeks, then 5 mg/week) | Resolution after 2-week therapy | [2] |
| 7 | 12/F | 16 months after recommencing HD; 5 months after a second transplant | Bullous lesions on the soft palate, then on the face, trunk, and extremities; subepidermal bullae with edematous dermal papillae; IF staining: IgG, C3, C1q in granular deposits on epidermal basement membrane. | Prednisolone (20 mg) on alternate days (Fail); plasma exchange (Fail); nephrectomy | Resolution within 1 month after nephrectomy | [5] |
| 8 | 12/M | 2 years after HD | Bullous, pruritic lesions around the AVF, then spread to trunk, thighs and oral mucous; subepidermal blister with eosinophils and neutrophils infiltration; direct IF: linear deposits of IgG and C3 at the DEJ; IgG and C3 on blister roof; indirect IF: IgG binding to the epidermal side of BMZ | Prednisolone (1 mg/kg QD), 0.05% clobetasol propionate; nephrectomy | Resolution after nephrectomy | [6] |
| 9 | 13/M | 2 months after discontinuation of IS therapy | Bullous lesions on trunk, extremities; subepidermal blister containing fibrin and scanty eosinophils infiltration; direct IF: linear C3 at the DEJ; indirect IF: anti-BMZ IgG binding to the epidermal surface; anti-BP180 IgG (+); anti-BP230 IgG (-) | Prednisolone (0.7 mg/kg, relapsed after reducing the dose), nephrectomy | Resolution | [7] |
| 10 | 15/F | 4 months after dialysis | Bullous lesions on the trunk, extremities and oral mucous; subepidermal blister with neutrophils and a mild eosinophils infiltration; direct IF: IgG and C3 at the DEJ | Prednisolone (1 mg/kg), azathioprine (100 mg) for a month | Resolution within a month | [8] |
| 11 | 15/F | 7 months after renal transplantation; 5 years after HD | Bullous lesions on face, lips, extremities; eosinophilic spongiosis, pauci-inflammatory subepidermal blister; direct IF: linear IgG and C3 deposits along the DEJ; Indirect IF: linear deposits of IgG at the DEJ | Prednisone (50 mg/day) for 2 weeks, then was reduced | Resolution after months | [9] |
| 12 | 27/M | 10 years after renal transplantation, 1 month after tapering IS | Bullous, pruritic lesions over trunk and extremities occurred concomitantly with hematuria; subepidermal blister with eosinophils infiltration in the superficial dermis; direct IF: linear IgG and C3 deposits along DEJ | Prednisolone (30 mg QD) | Resolution | [10] |
| 13 | 27/F | 1.5 months after taking levofloxacin, years after HD, renal rejection | Bullous, pruritic lesions on face, trunk, and upper extremities; ELISA: BP180 (+), BP230 (-); direct IF: linear C3 deposits along BMZ; indirect IF: anti-BMZ IgG binding to the epidermal surface | Discontinue levofloxacin, prednisone (60 mg/day) | Resolution | [11] |
| 14 | 33/M | 1 years after fistula placement | Bullous, pruritic lesions were around the fistula and spread to trunk and extremities; anti-BP180 IgG (+) | 0.05% clobetasol propionate (BID) | Resolution within 3 weeks | [12] |
| 15 | 34/F | 2 years after HD; 4 months after renal transplantation | Bullous lesions on upper limbs, thorax, and oral mucosa; eosinophils, lymphocytes and neutrophils infiltration; anti-BP180 IgG (+), anti-BP230 IgG (-) | Steroid and dapsone (relapse); prednisone, azathioprine and IVIG (all failed); nephrectomy | Resolution after nephrectomy | [13] |
| 16 | 35/F | 10 days after the addition to everolimus | Diffuse vesicles eruption; subepidermal blister; direct IF: linear deposits of IgG and C3 at the DEJ; BP180 (+); | Prednisone (Fail); reduce the dosage of everolimus | Immediate improvement after drug discontinuation and relapse after re-exposure | [14] |
| 17 | 39/M | 4 days after discontinuation of IS; 4 months after HD | Bullous lesions on extremities and trunk; eosinophils, lymphocytes, and neutrophils infiltration; direct IF: linear deposition of IgG and C3 along the DEJ; indirect IF: linear IgG at the BMZ and epidermal staining with IgG on NaCl-split skin | Prednisone (0.75 mg/kg/d); nephrectomy | Relapse 5 months though still using prednisone, resolved after nephrectomy | [15] |
| 18 | 42/F | 17 years after renal transplantation, together with acute rejection; relapse after discontinuation of IS therapy | Bullous lesions on neck, face, trunk, inferior extremities; anti-BP180 IgG (+) | Nephrectomy, prednisone (0.5/ mg/kg) | Resolution within one month | [16] |
| 19 | 43/M | 17 years after HD | Bullous, pruritic lesions, together with dry and rough pigmented skin, were on light-exposed areas and mucosa; mononuclear cells and eosinophils infiltration; circulating anti-BMZ IgG (+); direct IF: linear deposits of IgG and C3 at the DEJ; deposition of anti-BMZ antibody along the epidermal side of NaCl-separated skin. | Prednisone (50 mg QD), topical 0.1% diflucortolone valerate ointment | Resolution | [17] |
| 20 | 45/F | 2 months after withdrawal IS therapy; 10 months after renal allograft failed; 16 years after renal transplantation | Bullous lesions were mostly on extremities, also on trunk; eosinophils infiltration; IF: deposits of antibodies at the BMZ | Prednisone, azathioprine | Resolution | [18] |
| 21 | 46/M | 6-8 h after HD | Bullous lesions were widespread; anti-BP180/230 IgG (+) | Methylprednisolone (80 mg/day) and IVIG (400 mg/kg QD, 5 days) (Fail); dexamethasone solution prerinsing | Resolved by dexamethasone solution prerinsing | [19] |
| 22 | 47/F | 8 months after recommencing HD | Bullous lesions on arms, abdomen; eosinophils infiltration; direct IF: continuous C3 band at the basement membrane | Methylprednisolone (4 mg/day) (Fail); Methylprednisolone (12 mg/day) for 3 months (lesions cleared, then recurred after stopping treatment); nephrectomy | Lesions cleared but recurred after stopping drugs; resolution after nephrectomy | [20] |
| 23 | 48/M | 5 years after HD | Bullous lesions on the trunk | Methylprednisolone (60 mg/day) and topical halometasone (Fail); MTX (10 mg/week) | Rapid improve after taking MTX, but with side effects | [21] |
| 24 | 48/M | 13 years after HD; XLAS | Bullous, painful lesions on trunk, extremities, especially on palms and soles; anti-BP180 and anti-BP230 antibodies (+); neutrophils and eosinophils infiltration; BPDAI: 122 | Prednisone (55 mg/day) | Resolution | [22] |
| 25 | 49/M | 6 months after restarting HD; 8 years after renal transplantation | Bullous lesions on the limbs and soft palate; eosinophils infiltration; anti-BP180 IgG (+), anti-BP230 IgG (-) | Prednisone, azathioprine, cyclosporine A, and IVIG (All failed); nephrectomy | Resolution after nephrectomy | [13] |
| 26 | 50/M | 1 year after HD, 6 years after the second renal transplantation | Bullous lesions on hands, face, abdomen, oral mucosa; anti-BP180 IgG (+), anti-BP230 IgG (-); scant inflammatory cells infiltrate | Prednisone (80 mg) (Fail); nephrectomy | Resolution after nephrectomy | [23] |
| 27 | 52/F | 9 years after HD | Bullous, pruritic lesions overlying an erythematous base were around the fistula; direct IF: linear deposits of IgG, IgM, and C3 at basement membrane; lymphohistiocytic and eosinophils infiltrate; anti-nuclear antibodies (+) | Prednisone (30 mg QD) | Resolution | [24] |
| 28 | 52/M | 15 years after HD; 13 years after renal transplantation | Bullous, pruritic lesions on scalp, trunk, mucosa; subepidermal bullous disease; direct IF: linear IgG, C3, and C1q deposits along basement membrane; anti-BMZ antibody (+) | Topical clobetasol propionate, methylprednisolone, dapsone, plasmapheresis (all fail). | Died | [25] |
| 29 | 59/F | 9 years after renal transplantation | Bullous lesions on trunk; subepidermal blister; direct IF: IgG and C3 deposits at the DEJ; indirect IF: IgG along the epidermal side of NaCl-separated skin; anti-BP180 IgG (+), anti-BP230 IgG (-) | IV methylprednisolone (1 mg/kg/day), oral dapsone (100 mg/d), steroids | Resolution | [26] |
| 30 | 60/M | 16 months after HD; 12 months after discontinuing IS | Bullous lesions on extremities, oral mucosa; Anti-BP IgG (+); eosinophils infiltration | Prednisolone (40 mg/day); Nephrectomy | Resolution after nephrectomy | [27] |
| 31 | 62/M | 5 months after restarting HD; 6 months after renal rejection and IS discontinuation | Bullous, pruritic lesions on the lips, neck, trunk, upper extremities; anti-BMZ IgG (+); eosinophils infiltration | Prednisone (1 mg QD), azathioprine (50 mg/day), topical 0.05% clobetasol propionate (BID) | Resolution within 2 weeks | [28] |
| 32 | 63/M | 2 years after restarting HD; 4 years after renal transplantation | Bullous lesions all over the body; subepidermal bulla with eosinophils infiltration; direct IF: linear deposits of C3 along basement membrane; indirect IF: 1:80 titer for BP antibodies | Systemic corticosteroid | Resolution | [29] |
| 33 | 68/M | 4 years after HD; 16 months after renal transplantation | Bullous lesions on extremities; subepidermal blister; direct IF: linear IgG and C3 deposits at BMZ; anti-BP180 IgG (+) | Methylprednisolone (60 mg/day), MMF (2 g/day) | Resolution | [30] |
| 34 | 72/F | 2 days after creating vascular access by prosthetic graft | Bullous lesions around the graft site; eosinophils and neutrophils infiltration; leukocytosis; IF: linear deposition of IgG and complement. Immunoperoxidase staining: type IV collagen at the base of bulla | Graft removal 5 days after its placement, prednisone, tetracycline, and niacinamide | Resolved after graft removal but relapsed after the second operation | [31] |
| 35 | 73/M | 2 weeks after AVF; 4 years after HD | Bullous lesions around the AVF, without milia | Topical clobetasol propionate (BID) | Resolution within 4 weeks | [32] |
| 36 | 73/M | 8 years after HD | Bullous, pruritic lesions on trunk and extremities; mononuclear cells and a few eosinophils infiltration; BP180 antigen (-); BP230 antigen (+); direct IF: linear deposition of IgG and C3 at the DEJ; indirect IF: linear IgG and C3 on the epidermal side of split; | Topical 0.12% betamethasone valerate ointment containing 0.1% gentamicin (BID) | Resolution after 6 months | [33] |
| 37 | 75/M | Several months after changing the dialysis membrane | Bullous, pruritic lesions over trunk and extremities; anti-BP180 (+); anti-BP230 (+); eosinophils and lymphocytes infiltration | Topical clobetasol propionate (BID) for a week but was ineffective, then the dialysis membrane was replaced | 5 weeks after changing the membrane, lesions disappeared | [34] |
| 38 | 76/M | 2 years after HD | BP was diagnosed on clinical, immune-histological grounds | Topical clobetasol propionate (Fail), methotrexate (2.5 mg BIW after HD) | Died from methotrexate-induced pancytopenia | [35] |
| 39 | 76/M | 7 years after CRF | Bullous, pruritic lesions around fistula; eosinophils and lymphocytes infiltration; direct IF: linear deposits of IgG, IgM and C3 at basement membrane. | Prednisone (30 mg QD) | Resolution within 4 weeks | [24] |
| 40 | 76/M | Soon after the placement of the jugular catheter | Bullous, pruritic lesions around fistula, then to trunk, neck and face; eosinophils infiltration | Topical corticosteroids | Resolution | [36] |
| 41 | 77/M | 7 years after the start of HD | Bullous lesions on right hand (distal to arteriovenous fistula); subepidermal blistering with infiltration of eosinophils and lymphocytes in the upper dermis; direct IF: IgG and C3 deposits along BMZ; indirect IF (+) | ND | ND | [37] |
| 42 | 9/M | 5 years after renal transplant | Bullous, haemorrhagic lesions on the face, trunk, and extremities; subepidermal bullae with a light lymphocytic infiltration; direct IF: IgG and C3 at BMZ; indirect IF: IgG along the epidermal side of NaCl-separated skin. Immunoblotting: BP180 (+) | Corticosteroids | Resolution with graft atrophy | [38] |
| 43 | 17/F | 2 months after the diagnosis of chronic rejection | Bullous lesions on oral mucosa; anti-BMZ IgG (+) | Prednisolone (20mg on alternate days), plasma exchange, nephrectomy | Resolution after nephrectomy | [5] |
| 44 | 33/M | 5 years after renal transplantation | Bullous, pruritic lesions on trunk; anti-BP180 IgG (+); anti-BP230 IgG (+); eosinophils inf1iltration; | Prednisolone (50 mg/day) | ND | [39] |
| 45 | 34/F | 1 week after stopping tacrolimus; 6 years after renal transplantation | Bullous lesions on lower extremities, erosions on lower back, abdomen, and buccal mucosa; eosinophils infiltration; anti-BP180 (+) | Clobetasol 0.05% gel and ointment (Fail); clobetasol ointment, prednisone (80 mg/day) with magic mouth wash, 2 rituximab transfusions | Resolution | [23] |
| 46 | 46/M | 8 years after renal transplantation, BP onset together with renal graft rejection | Bullous lesions on face, trunk, extremities; direct IF: IgG and C3 linear deposits along DEJ; antinuclear antibody (+); anti-BP antigen antibody (+); minimal lymphocytes infiltration | High-dose steroids, restart mycophenolate | Resolution | [40] |
| 47 | 50/M | 6 years after renal transplantation | Bullous lesions; subepidermal blistering with eosinophils infiltration; direct IF: linear and continuous deposits of C3 on the DEJ; anti-BP180 IgG (+), anti-BP230 IgG (-) | Oral steroids and dapsone (50 mg/day) | Resolution after nephrectomy | [41] |
| 48 | 61/M | Suffering from ESKD for 1 year | Bullous lesions on the left foot; subepidermal blistering with moderate infiltration of eosinophils and lymphocytes; direct IF: linear deposits of IgG and C3 on the BMZ; anti-BP180 IgG (+) | Oral prednisolone (30 mg/day) | Resolution within a week | [42] |
| 49 | 63/M | 5 years after renal transplantation | Bullous lesions on neck, back, limbs, around nipples; skin biopsy was consistent with BP. | High-dose corticosteroid | Resolution | [43] |
| 50 | 65/M | 2 months after the addition to sirolimus | Bullous lesions on the trunk and limbs radix; subepidermal blistering with eosinophils infiltration; direct IF: linear deposits of C3 on the BMZ; anti-BP180 IgG (+) | Intravenous corticosteroid (Occasionally effective); Prednisone (little improvement) | Immediate improvement after drug discontinuation and relapse after re-exposure | [14] |
| 51 | 80/F | Time interval not described, but BP onset after ESKD | Erythematous-urticarial papules on the trunk, extremities; ELSA: anti-BP180 antibodies (+); direct IF: linear deposits of IgG on the DEJ; indirect IF: circulating antibodies linearly bind to the DEJ. | Prednisone (0.5mg/kg/day, fail), tralokinumab (600mg/2 weeks) | Resolution after treating with tralokinumab | [44] |

Cases 1-6 received peritoneal dialysis (PD), where cases 7-42 received hemodialysis (HD). The history of dialysis has not described in cases 43-50, but they all received renal transplantation. Case 51 was not on HD.

No., case number; BP, bullous pemphigoid; PD, peritoneal dialysis; HD, hemodialysis; ND, not described; CRF, chronic renal failure; QD, once a day; BID, twice a day; BIW, twice a week; IVIG, intravenous immunoglobulin; BMZ, basement membrane zone; IS, immunosuppressive; XLAS， X-linked Alport syndrome; BPDAI, BP disease area index; AVF, arteriovenous fistula; PN, pemphigoid nodularis; IF, immunofluorescent; DEJ, dermo-epidermal junction; MMF, mycophenolate mofetil;

**Supplement Table II. Other Subtypes of Pemphigoids diseases**

| **No.** | **Type** | **Age/sex** | **Latency** | **Characteristics** | **Therapy** | **Outcome** | **Ref** |
| --- | --- | --- | --- | --- | --- | --- | --- |
| 1 | EBA | 52/M | 5 years after putting on renal replacement therapy | Multiple wounds and small ulcers on hands, legs, and feet. Skin lesion biopsy confirmed diagnosis of inflammatory EBA | Prednisolone (5 mg/ day) | Mild improvement in the lesions | [45] |
| 2 | LAD | 13/M | 5 years after renal transplantation. | Bullous lesions on the face, trunk and lower limbs; subepidermal blister with infiltration of eosinophils and neutrophils; IF: linear IgA and C3 deposits along DEJ. | Steroids and dapsone. | Resolution | [46] |
| 3 | LAD | 27/F | 2 weeks after taking cefixime | Bullous lesions over extremities, then spread to trunk, oral and buccal mucosa; subepidermal blister with neutrophil-predominant and eosinophils infiltration; direct IF: linear IgA deposits along BMZ. | 0.05% topical clobetasol propionate (BID for 2 weeks) | Resolution | [47] |
| 4 | LAGBD^1^ | 43/M | Have ESKD secondary to hypertension (on HD) | Bullous lesions on the trunk and extremities; Sloughing of the conjunctiva and tongue; subepidermal split with eosinophils infiltration; direct IF: deposits of linear C3, equivocal IgG, and absent IgA along the BMZ; indirect IF with salt split skin: linear IgG and IgA at the roof of the induced blister; anti-BP180 IgG and IgA (+); anti-BP230 antibodies (-) | Prednisone (2 weeks, failed); rituximab (375 mg/m^2^/week for 4 weeks), MMF (500 mg BID), prednisone (80 mg) | Resolution after 4 rituximab infusions. Relapsed but resolved with 2 rituximab infusions. | [48] |
| 5 | LAD | 50/F | Chronic renal failure for >20 years and receives dialysis | Bullous lesions on the trunk and extremities; subepidermal blistering with a dense mixed cell dermal infiltration; direct IF: smooth linear deposition of IgA along the BMZ. | Prednisone (60 mg/day); IVIG (4 g/kg) | Resolution after IVIG therapy. | [49] |
| 6 | LAD^2^ | 53/M | ND | Bullous lesions over extremities, trunk; subepidermal blistering with inflammatory infiltrate; direct IF: granular deposition of IgA, C3, fibrinogen in dermal papillae, deposition of fibrinogen in a vascular pattern in papillary dermis | Pulse IV methylprednisolone; oral prednisone and dapsone | Resolution after 3 weeks | [50] |
| 7 | P200 pemphigoid | 70/M | ND | Bullous lesions on the trunk and extremities; subepidermal blister with neutrophils and eosinophils infiltration; direct IF: linear C3 deposits at the BMZ; indirect IF: anti-BMZ IgG at 1:20 tilter, which reacted with the dermal side on NaCl-split skin; immunoblotting: anti-laminin γ1, α3 and γ2 subunit of laminin-332 IgG (+) | Prednisolone (25 mg/ day), colchicine (0.5 mg/day) | Resolution | [51] |
| 8 | P200 pemphigoid & MMP | 61/M | 8 months after discontinuation of IS therapy | Bullous lesions on oral mucosa; direct IF: linear deposition of C3 at the BMZ; indirect IF: linear IgG deposits in the dermal side on NaCl-split skin; anti-γ2 subunit of laminin-332 IgG (+), anti-laminin γ1 antibodies (+) | Methylprednisolone, MMF, tacrolimus, | Resolution | [52] |
| 9 | MMP | 26/F | 1 years after renal transplantation | Ulcers on oral mucosa; lymphocytes infiltration; Nikolsky sign (+); subepithelial clefts with lymphocyte infiltration | Methylprednisolone (48 mg/day) | Resolution | [53] |

LAD, linear IgA dermatosis; EBA, epidermolysis bullosa acquisita; AVF, arteriovenous fistula; PN, pemphigoid nodularis; MMP, mucous membrane pemphigoid.

^1^This patient had a mixed pattern of LAD and BP, referred to as linear IgA/IgG bullous dermatosis (LAGBD)

^2^This patient had a mixed pattern of LAD and dermatitis herpetiformis

**Reference**

1. Takeichi, S., et al., Brunsting-Perry type localized bullous pemphigoid, possibly induced by furosemide administration and sun exposure. Eur J Dermatol, 2009. 19(5): p. 500-3.

2. Morimoto, K., et al., Bullous pemphigoid in patients receiving peritoneal dialysis: a case series and a literature survey. Ren Fail, 2021. 43(1): p. 651-657.

3. Giunzioni, D., Development of Bullous Pemphigoid after Tenckhoff Catheter Placement in a Peritoneal Dialysis Patient. Case Rep Dermatol, 2020. 12(1): p. 42-46.

4. Michelerio, A. and C. Tomasini, Blisters and Milia around the Peritoneal Dialysis Catheter: A Case of Localized Bullous Pemphigoid. Dermatopathology (Basel), 2022. 9(3): p. 282-286.

5. Feehally, J., et al., An antibody-mediated bullous skin eruption caused by chronic renal allograft rejection. Transplantation, 1982. 34(5): p. 295-6.

6. Mammen, C., C.T. White, and J. Prendiville, Childhood bullous pemphigoid: A rare manifestation of chronic renal allograft rejection. J Am Acad Dermatol, 2011. 65(1): p. 217-219.

7. Davis, R.F., et al., Bullous pemphigoid associated with renal transplant rejection. Clin Exp Dermatol, 2011. 36(7): p. 824-5.

8. Peruzzo, J., L. Dias Pinheiro Dantas, and M. Zampese, Bullous pemphigoid associated with chronic renal allograft rejection. J Am Acad Dermatol, 2013. 68(6): p. e192-e193.

9. Morelli, J.G. and W.L. Weston, Childhood immunobullous disease following a second organ transplant. Pediatr Dermatol, 1999. 16(3): p. 205-7.

10. Liaw, T.-Y., et al., Bullous pemphigoid in a chronic renal allograft rejection patient: a case report and review of the literature. Dermatol Sin, 2011. 29(3): p. 94-97.

11. Miao, J., L.E. Gibson, and I.M. Craici, Levofloxacin-Associated Bullous Pemphigoid in a Hemodialysis Patient After Kidney Transplant Failure. Am J Case Rep, 2022. 23: p. e938476.

12. Osipowicz, K., et al., Development of bullous pemphigoid during the haemodialysis of a young man: case report and literature survey. Int Wound J, 2017. 14(1): p. 288-292.

13. Rosique López, F., et al., Bullous pemphigoid and chronic kidney graft rejection. J Eur Acad Dermatol Venereol, 2017. 31(11): p. e508-e510.

14. Atzori, L., et al., Bullous pemphigoid induced by m-TOR inhibitors in renal transplant recipients. J Eur Acad Dermatol Venereol, 2015. 29(8): p. 1626-30.

15. Rodríguez-Caruncho, C., et al., Bullous pemphigoid associated with chronic renal allograft rejection: resolution after transplantectomy. J Am Acad Dermatol, 2011. 65(3): p. e89-e90.

16. Sartor, E., et al., Bullous pemphigoid and renal graft rejection: is there a causative link? Eur J Dermatol, 2020. 30(4): p. 441-442.

17. Sato, M., et al., Bullous pemphigoid masquerading as porphyria cutanea tarda in a patient on haemodialysis. Br J Dermatol, 1993. 129(5): p. 642-643.

18. Abdul Salim, S., et al., New diagnosis of bullous pemphigoid after withdrawal of immunosuppressive therapy in a failed renal transplant recipient on hemodialysis. Hemodial Int, 2018. 22(2): p. E26-e32.

19. Suo, H., et al., Development of generalized bullous lesions after hemodialysis with polysulfone membrane dialyzer. J Dermatol, 2020. 47(4): p. e119-e120.

20. Tessari, G., et al., Bullous eruption during chronic renal allograft rejection. Dermatology, 2002. 204(4): p. 307-8.

21. Liu, H., et al., Combined acute interstitial pneumonitis and pancytopenia induced by low-dose methotrexate in a hemodialysis patient treated for bullous pemphigoid. An Bras Dermatol, 2015. 90(3 Suppl 1): p. 43-5.

22. Yamawaki, M., et al., Bullous Pemphigoid in X-linked Alport Syndrome. Intern Med, 2023. 62(16): p. 2375-2379.

23. Hodges, W.T., et al., Bullous pemphigoid with prominent mucosal involvement in the setting of renal allograft rejection. JAAD Case Rep, 2024. 44: p. 53-57.

24. Pardo, J., et al., Localized bullous pemphigoid overlying a fistula for hemodialysis. J Am Acad Dermatol, 2004. 51(2 Suppl): p. S131-2.

25. Chen, T.J., et al., Bullous pemphigoid in a renal transplant recipient: a case report and review of the literature. Am J Clin Dermatol, 2009. 10(3): p. 197-200.

26. Cavaliere, G., et al., Bullous pemphigoid in a renal transplant recipient. Eur J Dermatol, 2014. 24(3): p. 383-4.

27. Barata, R., et al., A rare presentation of kidney allograft intolerance syndrome: Bullous pemphigoid. Nephrol Ther, 2021. 17(7): p. 547-551.

28. Bao, C., et al., Bullous pemphigoid in kidney transplant rejection: A case report and literature review. Australas J Dermatol, 2022. 63(1): p. e80-e83.

29. Simon, C.A. and R.K. Winkelmann, Bullous pemphigoid and glomerulonephritis. Report of four cases. J Am Acad Dermatol, 1986. 14(3): p. 456-63.

30. De Simone, C., et al., Bullous pemphigoid in a transplant recipient: is this a sign of allograft rejection? Eur J Dermatol, 2012. 22(2): p. 280-1.

31. Freeman, B.D. and B.G. Rubin, Bullous pemphigoid after prosthetic vascular graft placement. Surgery, 1998. 124(1): p. 112-3.

32. Yesudian, P.D., et al., Trauma-induced bullous pemphigoid around venous access site in a haemodialysis patient. Clin Exp Dermatol, 2002. 27(1): p. 70-72.

33. Kamada, N., et al., A case of bullous pemphigoid in a patient on hemodialysis. J Dermatol, 1998. 25(4): p. 246-9.

34. Sodemoto, K., et al., Development of bullous pemphigoid after change of dialysis membrane. Hemodial Int, 2014. 18(2): p. 525-8.

35. Seneschal, J., I. Héliot-Hostein, and A. Taieb, Pancytopenia induced by low-dose methotrexate in a haemodialysis patient treated for bullous pemphigoid. J Eur Acad Dermatol Venereol, 2007. 21(8): p. 1135-6.

36. Jacobs, L., et al., Severe Bullous Pemphigoid Onset after Jugular Catheter Placement in a Patient on Hemodialysis. Case Rep Nephrol Dial, 2022. 12(2): p. 138-144.

37. Jang, J.W., et al., A Case of Localized Bullous Pemphigoid Associated with an Arteriovenous Fistula. Indian J Dermatol, 2020. 65(6): p. 547-548.

38. Yamazaki, S., et al., Childhood bullous pemphigoid associated with chronic renal allograft rejection. Br J Dermatol, 1998. 138(3): p. 547-8.

39. Mohaghegh, F. and R.S. Khalili Tembi, Bullous Pemphigoid in a Renal Transplant Recipient, A Case Report and Review of the Literature. Iran J Kidney Dis, 2020. 14(5): p. 415-417.

40. Sofi, A.A., et al., Bullous pemphigoid associated with acute renal allograft rejection. Transplantation, 2010. 89(3): p. 368-9.

41. Devaux, S., et al., Chronic renal graft rejection-associated bullous pemphigoid: A cross-reactive immune response? Acta Derm Venereol, 2011. 91(1): p. 82-3.

42. Oka, M., Localized Bullous Pemphigoid in a Patient with Acquired Reactive Perforating Collagenosis. Case Rep Dermatol, 2023. 15(1): p. 1-4.

43. Koratala, A., et al., The skin-kidney connection: bullous pemphigoid associated with acute allograft rejection and membranous nephropathy. Clin Case Rep, 2018. 6(2): p. 432-433.

44. Maglie, R., et al., Rapid and sustained response to tralokinumab in a patient with severe bullous pemphigoid and end-stage kidney disease. Clin Exp Dermatol, 2024. 49(2): p. 161-163.

45. Umar, R.M., A.A. Khan, and W. Ahmad, Epidermolysis Bullosa Acquisita in a patient with End Stage Renal Disease: Case study. J Pak Med Assoc, 2023. 73(2): p. 405-406.

46. Kumar, G. and H.K. AlHadhrami, Bullous lesions in a young adolescent postrenal transplant. Saudi J Kidney Dis Transpl, 2020. 31(2): p. 553-555.

47. Sil, A., et al., Cefixime-induced linear IgA bullous dermatosis: a rare cause of mucocutaneous bullous eruption in a patient on hemodialysis. Int J Dermatol, 2022. 61(6): p. 763-765.

48. Nedosekin, D., et al., Immunologic overlap in a case of linear IgG/IgA bullous dermatosis responsive to rituximab. JAAD Case Rep, 2021. 9: p. 57-60.

49. Khan, I.U., K.C. Bhol, and A.R. Ahmed, Linear IgA bullous dermatosis in a patient with chronic renal failure: response to intravenous immunoglobulin therapy. J Am Acad Dermatol, 1999. 40(3): p. 485-8.

50. Bhat, Z.Y., et al., Bullous Dermatosis in an End-Stage Renal Disease Patient: A Case Report and Literature Review. Case Rep Nephrol, 2016. 2016: p. 6713807.

51. Kamata, M., et al., Anti-laminin γ1 pemphigoid accompanied by autoantibodies to laminin α3 and γ2 subunits of laminin-332. JAMA Dermatol, 2013. 149(12): p. 1437-9.

52. Mitate, E., et al., Concurrence of autoantibodies to both laminin γ1 and γ2 subunits in a patient with kidney rejection response. Acta Derm Venereol, 2013. 93(1): p. 114-5.

53. Zisis, V., et al., Alport Syndrome and Oral Mucous Membrane Pemphigoid: An Interesting Case. Cureus, 2023. 15(7): p. e41519.
